# A Genome-Wide Study of Cytogenetic Changes in Colorectal Cancer Using SNP Microarrays: Opportunities for Future Personalized Treatment

**DOI:** 10.1371/journal.pone.0031968

**Published:** 2012-02-20

**Authors:** Farzana Jasmine, Ronald Rahaman, Charlotte Dodsworth, Shantanu Roy, Rupash Paul, Maruf Raza, Rachelle Paul-Brutus, Mohammed Kamal, Habibul Ahsan, Muhammad G. Kibriya

**Affiliations:** 1 Department of Health Studies, The University of Chicago, Chicago, Illinois, United States of America; 2 Department of Human Genetics, The University of Chicago, Chicago, Illinois, United States of America; 3 Department of Medicine, The University of Chicago, Chicago, Illinois, United States of America; 4 Comprehensive Cancer Center, The University of Chicago, Chicago, Illinois, United States of America; 5 Department of Pathology, Bangabandhu Sheikh Mujib Medical University (BSMMU), Dhaka, Bangladesh; Ohio State University Medical Center, United States of America

## Abstract

In colorectal cancer (CRC), chromosomal instability (CIN) is typically studied using comparative-genomic hybridization (CGH) arrays. We studied paired (tumor and surrounding healthy) fresh frozen tissue from 86 CRC patients using Illumina's Infinium-based SNP array. This method allowed us to study CIN in CRC, with simultaneous analysis of copy number (CN) and B-allele frequency (BAF) - a representation of allelic composition. These data helped us to detect mono-allelic and bi-allelic amplifications/deletion, copy neutral loss of heterozygosity, and levels of mosaicism for mixed cell populations, some of which can not be assessed with other methods that do not measure BAF. We identified associations between CN abnormalities and different CRC phenotypes (histological diagnosis, location, tumor grade, stage, MSI and presence of lymph node metastasis). We showed commonalities between regions of CN change observed in CRC and the regions reported in previous studies of other solid cancers (e.g. amplifications of 20q, 13q, 8q, 5p and deletions of 18q, 17p and 8p). From Therapeutic Target Database, we identified relevant drugs, targeted to the genes located in these regions with CN changes, approved or in trials for other cancers and common diseases. These drugs may be considered for future therapeutic trials in CRC, based on personalized cytogenetic diagnosis. We also found many regions, harboring genes, which are not currently targeted by any relevant drugs that may be considered for future drug discovery studies. Our study shows the application of high density SNP arrays for cytogenetic study in CRC and its potential utility for personalized treatment.

## Introduction

Colorectal cancer (CRC) is a common malignancy in developed countries. In the USA it is the second highest cause of cancer-related deaths, with an estimated 102,900 new cases occurring during 2010 [1], [2]. CRC is typically much less common in developing countries of the world, including Southeast Asia; however, rates are on the rise, perhaps due to aging populations, smoking, changes in diet and a lack of screening programs [1]. In the South Asian population, patients tend to present with CRC at a younger age and typically at later stage [3], [4].

Cancer cells are characterized by cytogenetic abnormalities that can be used to define specific disease entities and their prognostic and predictive markers. In CRC, chromosomal abnormalities occur in a non-random pattern along the pathway from adenoma to carcinoma and then to advanced lesions and the formation of metastasis [5]–[8]. There are three known pathways in CRC pathogenesis: chromosomal instability (CIN), microsatellite instability (MSI), and the CpG island methylator phenotype (CIMP) pathways [9]. These three pathways are closely related and tumors occasionally exhibit features of multiple pathways. Most cases of CRC arise through the CIN pathway: for example, via structural rearrangements, amplifications and deletions [10], with copy number (CN) variation being a common finding [11], [12]. Some of the believed consequences of CIN are loss of tumor suppressor genes and amplification of oncogenes in the affected regions. In contrast, MSI is less common and is more likely to be associated with hereditary CRC and a better prognosis [8], [13]. CIN and MSI are thought to involve two separate pathways in the development of CRC [6], [10].

Chromosomal abnormalities in CRC have been studied by multiple groups using either comparative genomic hybridization (CGH) or array comparative genomic hybridization (aCGH) [5]–[8], [10]–[12], [14]–[22]. This has led to the discovery of many chromosomal aberrations, including gains and losses, portraying a complex picture of disease progression. Particularly common findings are gains in 20q, 13q, 7p, and 8q and losses in 17p, 18q, 8p, 4q, and 5q [23]–[29]. High-density single nucleotide polymorphism (SNP) arrays are an alternative and advantageous method for the analysis of chromosomal abnormalities. This is because a higher resolution can be achieved alongside simultaneous analysis of loss of heterozygosity (LOH) and CN variation [30]. To our knowledge, there are only a few published cytogenetic studies in CRC performed using relatively low-density SNP arrays [23], [29], [31]–[35], and these studies plead a strong case for their use. In 2007, Andersen et al., using a SNP array (Affymetrix 10 K array), found copy neutral LOH (cnLOH) as a common occurrence in CRC [23]. Middeldorp et al. genotyped FFPE tissues from 19 *MUTHY*-associated CRC patients using Illumina's Golden Gate genotyping and found similar changes mainly in 17p, 18q, 15q and 6p region [34]. CGH or aCGH are unable to detect the occurrence of cnLOH. The consequences of such lesion during human development and cancer have been recently reviewed [36]. SNP arrays therefore capture more detailed information regarding the underlying possible mechanism of the abnormalities detected. Gaasenbeek et al. [31] compared SNP array analysis on the Affymetrix platform (10 K Xba131) with CGH on 45 unselected CRC cell lines and found many abnormalities that were apparent using the SNP array but were undetected in CGH, confirming the advantage of using a SNP array platform. Lips et al. compared 4 FFPE CRC tissue using Illumina's GoldenGate SNP array (containing 5,861 probes) with DNA from leukocyte, normal FFPE and fresh frozen tissue from the same cases and found comparable results [32]. Furthermore, Lips et al. [29] used Affymetrix 10 K SNP array for 78 fresh frozen rectal tumor tissues to show the difference in CIN in adenoma and carcinoma. They found five specific chromosomal aberrations that could discriminate between adenoma and carcinoma. Sheffer et al. [35] used the Affymetrix 50 K XbaI SNP array to correlate CN variations in CRC with gene expression (using Affymetrix U133A), disease progression and survival outcomes. They tested 130 SNP array profiles from different types of tissue (normal, adenoma, different stages CRC and metastasis).They found deletions of 8p, 4p and 15q to be associated with progression of CRC and poor survival outcome.

In contrast to the previous studies, with paired and much larger sample size from a homogenous group of 86 CRC patients, our present study aimed to detect chromosomal aberrations using Illumina's Infinium based high-density (610 k and 300 k) SNP array. We analyzed the data for correlations between CIN and various histopathological and clinical parameters. Furthermore, we identified genes harbored within the affected regions which may contribute to tumor development or may have a protective effect on tumor metastasis. Using the Therapeutic Targets Database (TTD) we matched the genes in the amplification and deletion regions with drugs that are either already approved or are in stages of drug development for targeting these genes. With this novel interrogation, it is possible to identify appropriate drugs for patients exhibiting these particular aberrations and therefore tailor treatment to the individual. Our study, to our knowledge, is the first to report the findings from the use of a high density SNP array platform on a paired and much larger sample size to analyze chromosomal abnormalities in relation to tumor type, location, grade, stage and sex. Our study is also unique in correlating the altered genes with drugs targeting the genes for potential identification of personalized therapy. Finally, our study was conducted in an understudied Southeast Asian population with CRC.

## Results

**Figure 1:**
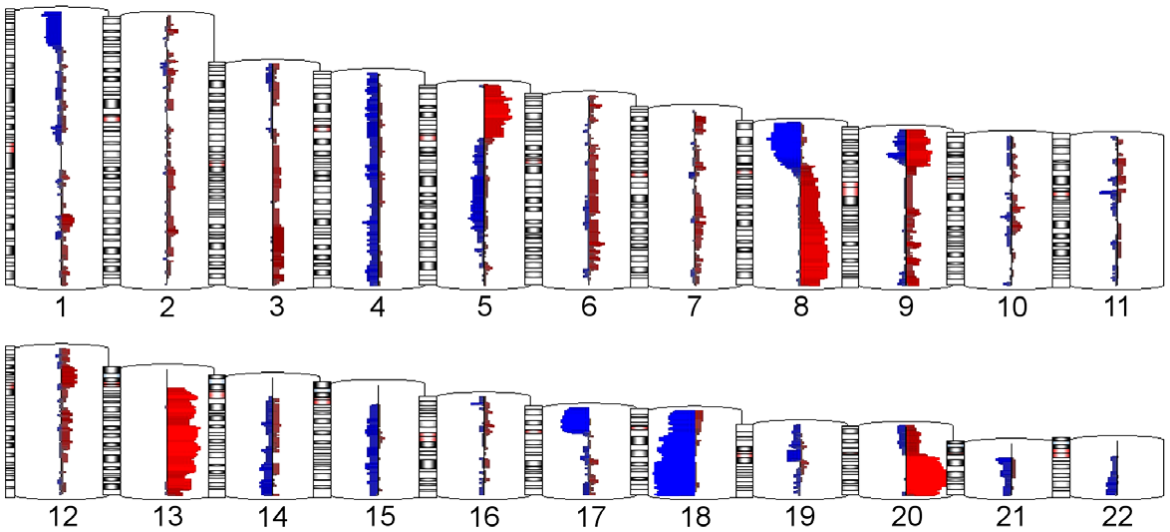
Amplified/deleteted regions observed in this study's CRC tissue samples. Amplifications (mean CN≥2.5) are shown in red, and deletions (mean CN≤1.5) are shown in blue. The horizontal size of the bar representation how many samples (at least 9 samples, 10%) exhibited the amplification/deletion.

Patient characteristics and histopathological data are presented in Table 1. Paired CN analysis of the SNP and Copy Number Variation (CNV) probe data from the 86 CRC tissues and their corresponding surrounding healthy colonic tissue samples revealed a total of 10,878 genomic segments (autosomes only) with amplification (1501), deletion (1630) and no CN change (7747). By “amplification” we mean an increase in the number of copies of a specific DNA fragment and by “deletion” we mean a loss of part of the DNA from a chromosome (http://www.ornl.gov/sci/techresources/Human_Genome/glossary/). The chromosomal locations of these amplifications (in red) or deletions (in blue) found in the CRC samples are shown in Figure 1. The width of the bar is proportional to the number of samples showing the CN change. For example, amplification was most frequently found in 20q13.2 region (21 of 86 CRC samples) and deletion was most frequently found in 18q21 region (19 of 86 CRC samples). In comparison to corresponding healthy tissue we found amplification in the following cytoband regions of the CRC tissue: chromosome 20 (q11, q12, q13, p11), chromosome 13 (q12, q13, q14, q21, q22, q24, q32, q33), chromosome 8 (q12, q21, q22, q23, q24), chromosome 5 (p12, p13, p14, p15) and chromosome 9 (p21, p22, p23, p24). Similarly, deletions were found in chromosome 18 (q21, q22, q23, q11, q12, p11), chromosome 8 (p21, p22, p23, p12), chromosome 17 (p11, p12, p13), chromosome 9p21 and chromosome 1p36. Most of these findings are also reported by other authors [29], [33], [35].

**Table 1:** Patient and tumor characteristics of 86 cases of CRC stratified by MSI status.

		MSI CRC	MSS CRC	p-value
Sex	male	16	34	0.343
	female	8	28	
Age		45.21 (SD12.27)	45.13 (SD15.14)	0.982
BMI		20.17 (SD 2.88)	21.58 (SD3.40)	0.077
Family history	yes	3	2	0.130
	no	21	60	
Location	left	13	54	**0.003**
	right	11	8	
Histopathology	Adenocarcinoma	21	54	1.000
	Mucinous adenocarcinoma	3	8	
Prop of tumor cells (%)		74.6 (SD6.24)	71.2 (SD6.48)	**0.029**
Lymph node involvement	No	14	27	0.239
	Yes	10	35	
Stage	stage-1	5	11	0.444
	stage-2	9	16	
	stage-3	10	35	
Grade	low	17	50	0.388
	high	7	12	

### Types of identified cytogenetic abnormalities

Unlike CGH, which provides only CN data, SNP chips provide information about both CN and BAF. The BAF refers to a measure of “allelic composition”, i.e., the relative contribution to the signal from the B allele. In other words, BAF for a sample shows the theta value for a SNP corrected for cluster position. Therefore, the BAF values for a particular locus for an individual who is homozygous for the B allele, homozygous for the A allele or heterozygous for AB are 1, 0 or 0.5 respectively. BAF offers two major advantages to the use of high density SNP array platforms in cytogenetic studies, as described below with illustration from relevant findings from our study.

**Figure 2:**
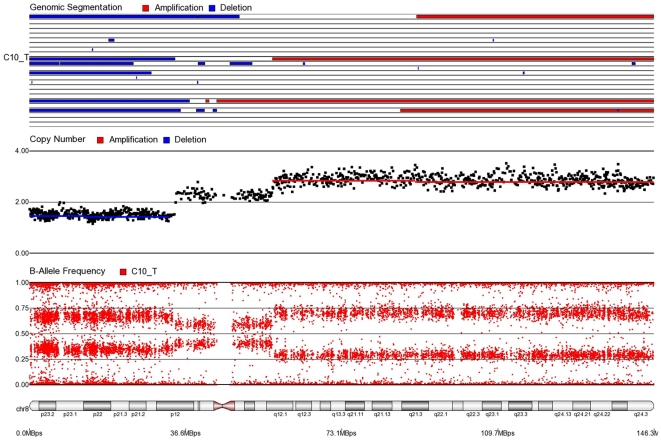
Mosaic monoallelic amplification and deletion in chromosome 8 for CRC tissue sample C10_T. In 8q (where BAF = 0.34, 0.66 and CN = 2.9), approximately 90% of cells have monoallelic amplifications (AAB or ABB). In 8p (where BAF = 0.35, 0.65 and CN = 1.55), approximately 40% of cells have monoallelic deletions (A_ or B_).

**Figure 3:**
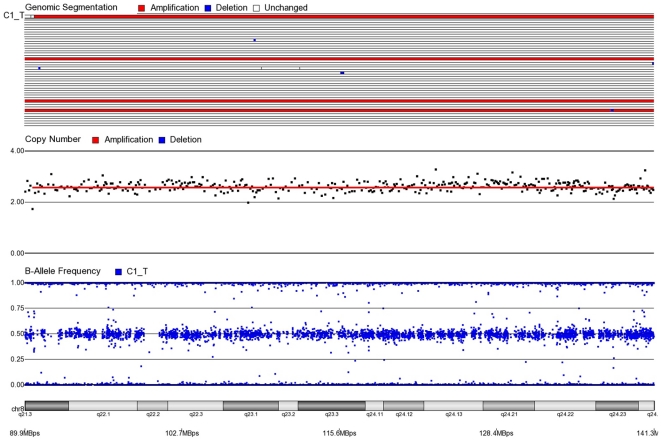
Mosaic biallelic amplification in chromosome 8q for CRC tissue sample C1_T. BAF = 0.5 and CN = 2.6. Approximately 30% of cells have biallelic amplification (ABAB).

**Figure 4:**
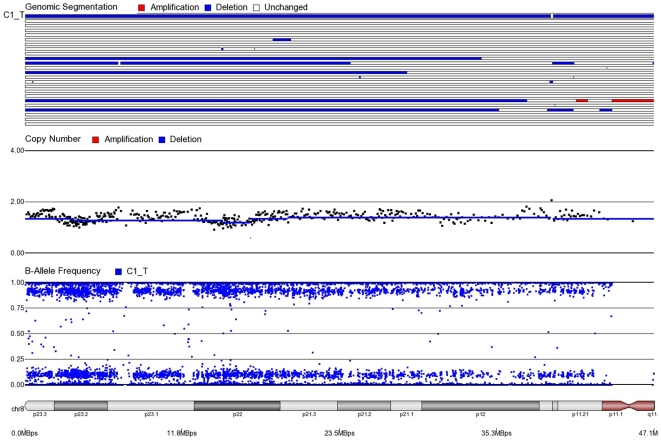
Mosaic monoallelic deletion in chromosome 8p for CRC tissue sample C1_T. BAF = 0.17, 0.83 and CN = 1.2. Approximately 80% cells have mono-allelic deletion (A_ or B_).

**Figure 5:**
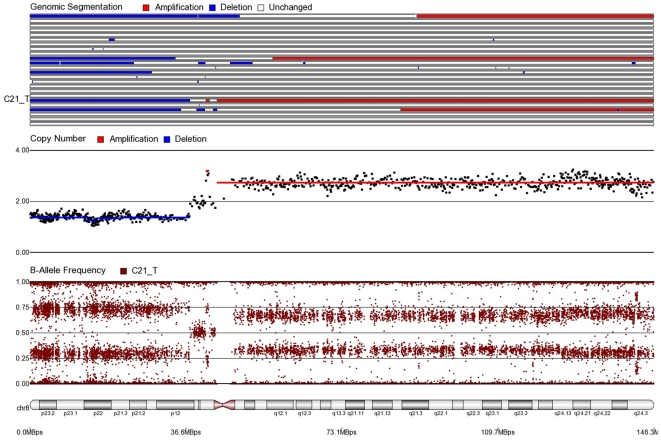
Mosaic monoallelic amplification and deletion in chromosome 8 for CRC tissue sample C21_T. In 8q (where BAF = 0.38, 0.62 and CN = 2.65), approximately 65% of cells have monoallelic amplifications (AAB or ABB). In 8p (where BAF = 0.26, 0.74 and CN = 1.35), approximately 65% of cells have monoallelic deletions (A_ or B_).

**Figure 6:**
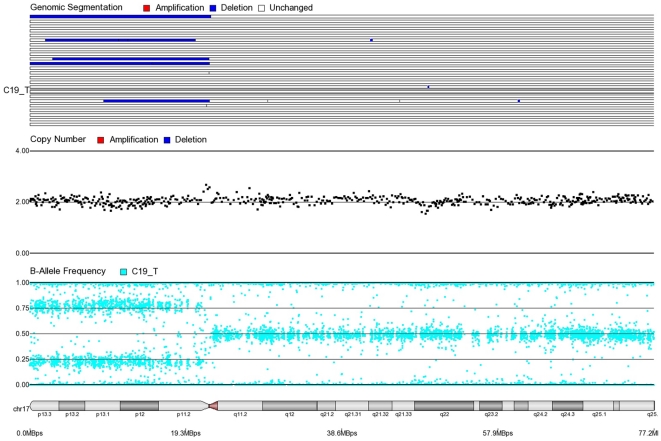
Mosaic cnLOH in chromosome 17 for CRC tissue sample C17_T. Note that 17p shows the normal heterozygote pattern in all cells (BAF = 0.5 and CN = 2) but approximately 50% of cells show cnLOH (AA or BB) in 17q (BAF = 0.25, 0.75 and CN = 2).

**Figure 7:**
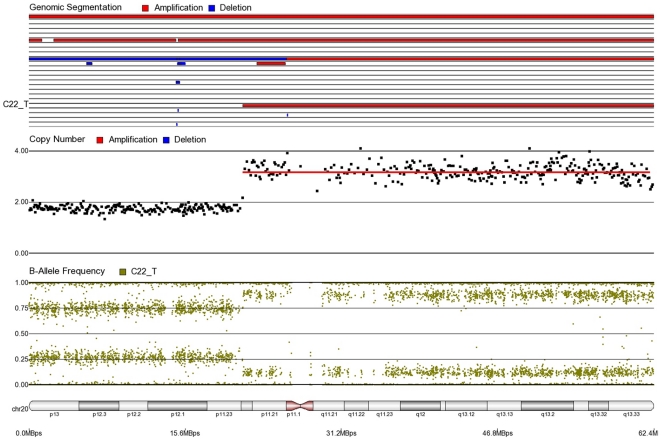
Mosaic isotrisomy in chromosome 20q of CRC tissue sample C22_T.

First, mosaicism or proportions of different cell populations (in this case, tumor cells) can be obtained from a back calculation using the formula described in our “Statistical Methods” section. In other words, the cytogenomic data derived from such high density SNP chips can also provide information regarding admixture of normal and chromosomally abnormal cells in the studied DNA sample. Figures 2–6 illustrate this in our study. In each of the figures, the upper panel shows the genomic segmentation in different samples (where each row represents a sample, amplification is shown in red, deletion in blue and normal CN without color); the second panel shows the CN in linear scale for a single sample marked in the first and third panel; the third panel shows the BAF of the same sample; and the bottom or fourth panel shows the corresponding chromosomal location with cytoband information. Figure 2 shows that almost the entire 8q arm of sample C_10 had amplification (CN 2.9) with splitting of heterozygote (AB) pane of BAF at ∼0.34 (representing AAB) and 0.66 (representing ABB) indicating mono-allelic amplification in ∼90% cells (in this case cancer cells) giving rise to a mixture of cells. The formula for calculation of mosaicism is shown in “Material and Method” section. It may be noted that if 100% of the cells had similar change, then theoretically we would expect CN = 3 and BAF to be 0.33 and 0.67 (a typical picture of trisomy). The same sample shows deletion in 8p in ∼45% cells. Figure 3 shows amplification in the same 8q arm in another sample, C_1. In contrast to Figure 2, however, BAF in this case does not show any split at all despite increase in CN suggesting bi-allelic amplification in ∼30% of the cell population. BAF, in this case looks absolutely like normal cell, but the CN data clearly shows amplification. Only the combination of CN and BAF data could suggest the possible pathology. If 100% cells had such change, we would expect CN = 4 and BAF = 0.5 (i.e., no splitting). Figure 4 shows chromosome 8p of sample C_1 and clearly shows mono-allelic deletion (e.g. A_ or _B) in ∼80% cells. If 100% cells had such mono-allelic deletion, we would expect CN = 1 and splitting of BAF to 0.0 and 1.0 (a typical picture of complete LOH due to mono-allelic loss). Figure 5 shows mono-allelic deletion of 8p and mono-allelic amplification of 8q both in one sample (C_21). The CN and the BAF splitting suggest this deletion-amplification in ∼65% cell population. Comparison of the deletion events in Figure 4 and Figure 5 clearly show the advantage of analyzing BAF data and CN data together to obtain the proportion of abnormal cells in the tumor sample. Figure 5 also clearly shows that splitting of AB pane in BAF plot may be found both in deletion and amplification states emphasizing the importance of combined data (CN and BAF) for interpreting the cytogenetic change seen in tumor samples. Figure 6 shows an example of splitting of AB pane in BAF data in chromosome 17p without any change in CN status (may be called cnLOH). This type of change is possible in case of acquired UPD or isodisomy. In this case (sample C_19) ∼50% cells show cnLOH in 17p arm only whereas the entire 17q arm is normal. It may be noted that this type of abnormality can not be detected by aCGH. Figure 7 shows an example of monoallelic amplification superimposed on cnLOH in chromosome 20q. A summary of the combination of changes in CN status and BAF and their possible explanation for the mechanism of cytogenetic abnormality is presented in Table 2.

**Table 2:** Interpretation of CN and BAF in autosomes for possible underlying mechanism of chromosomal abnormality.

Copy Number (CN)	Heterozygote pane in B-Allele Frequency (BAF)	Possible explanation	Example
Unchanged	No split	Normal	Figure 6 (chr 17q)
Amplified	Moderate split	Mono-allelic Amplification	Figure 2 (chr 8q)
Amplified	Large split	Mono-allelic amplification superimposed on cnLOH	Figure 7 (chr 20q)
Amplified	No split	Bi-allelic amplification	Figure 3 (chr 8)
Deleted	Large split	Mono-allelic deletion	Figure 4 (chr 8p)
Deleted	Moderate split	Mono-allelic deletion in small proportion of cells	Figure 11 (chr 6q)
Unchanged	Moderate split	cnLOH	Figure 6 (chr 17p), Figure 9 (chr 6)

**Figure 8:**
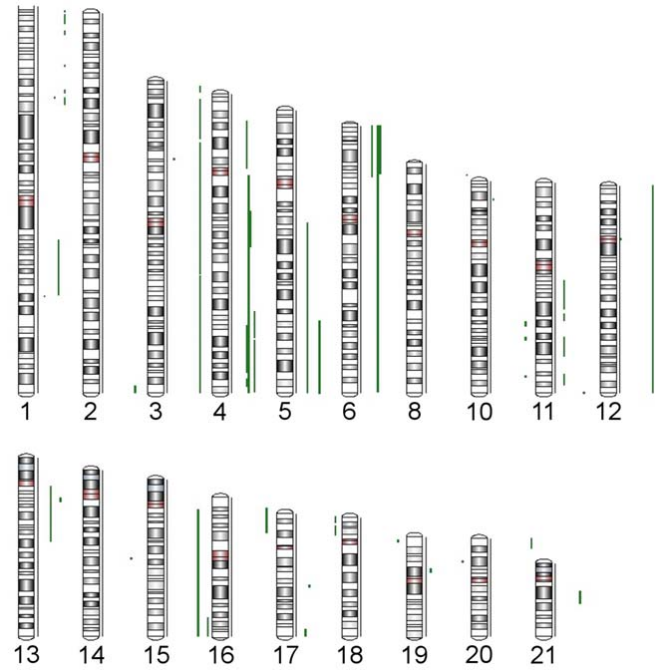
Regions of cnLOH found in this study's CRC samples. Each vertical green line shows the occurrence copy-neutral LOH in one sample.

**Figure 9:**
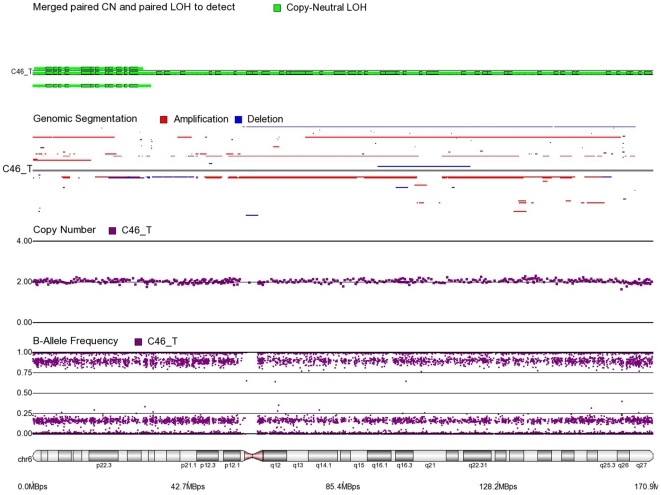
Mosaic cnLOH in chromosome 6 of CRC tissue sample C46_T. The cnLOH occurs in the entire chromosome.

**Figure 10:**
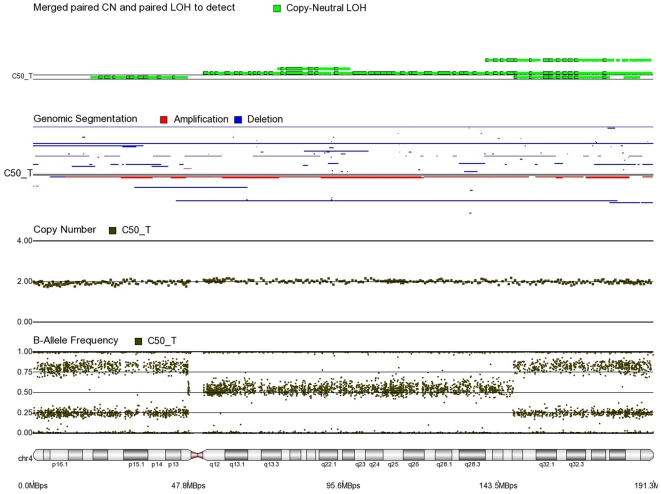
Mosaic cnLOH in chromosome 4 of CRC tissue sample C50_T. The cnLOH occurs in all of 4p and parts of 4q where BAF = 0.25, 0.75 and CN = 2. The portion of 4q where BAF = 0.5 and CN = 2 shows normal heterozygote patterns in all cells.

**Figure 11:**
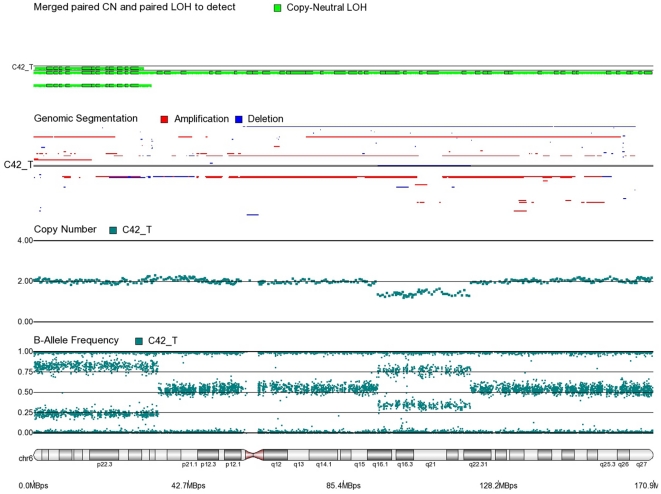
Mosaic cnLOH in chromosome 6 of CRC tissue sample C42_T. The cnLOH occurs in 6p where BAF = 0.25, 0.75 and CN = 2. The portion of 6q where CN = 1 is mosaic monosomy.

Second, with the use of high density SNP arrays, possible mechanisms such as mono-allelic or bi-allelic amplification/deletion, as well as cnLOH (or acquired UPD-like changes) can be detected, as illustrated by Figures 8–10. We used CN≥2.5 or ≤1.5 to mark a segment as amplification or deletion. So the software would consider any segment with CN between 1.6 and 2.4 as copy neutral, but to be conservative we considered only the regions which had CN 1.9–2.1 as copy neutral. The ideogram in Figure 8 shows the regions (in green) where we detected possible cnLOH in a number of cases. Each vertical line represents a sample. While some of these regions were reported previously [23], we identified several novel abberations in CRC (chromosome 8p, 10, 11, 12, 13, 14, 16p, 17q, 19p, 21q). The cnLOH can be found in the entire chromosome (Figure 9), in parts of the chromosome (Figure 10, entire p-arm and telomeric end of q-arm) or it may also be found in combination with deletion in another part of the chromosome (Figure 11, cnLOH in part of chromosome 6p and deletion in part of chromosome 6q).

### Association of cytogenetic abnormalities with tumor characteristics

**Figure 12:**
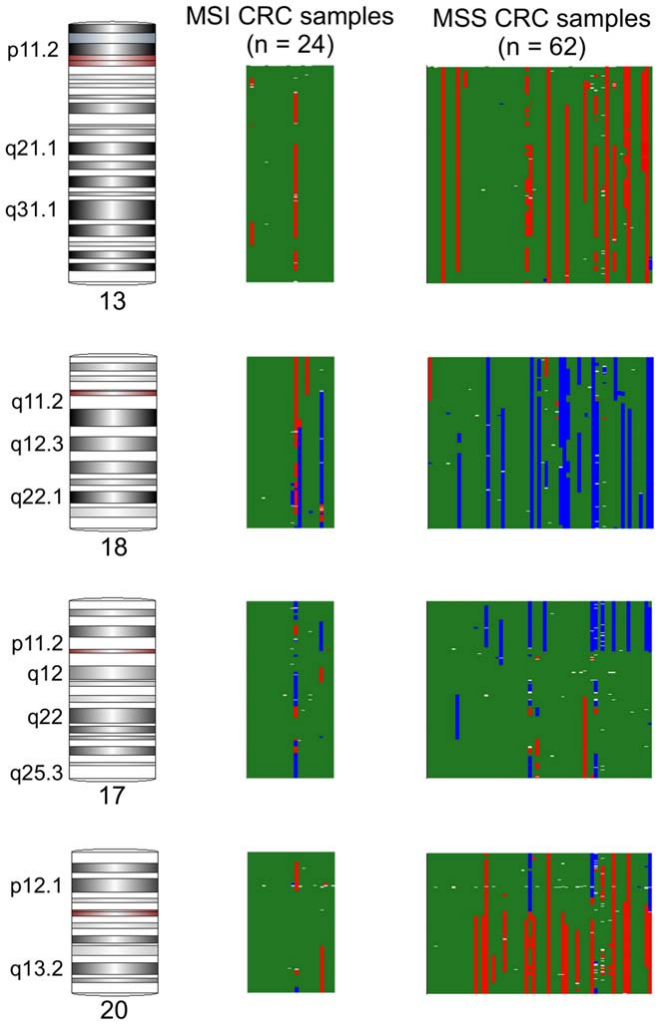
Genomic regions where the frequency of amplification or deletion is significantly different between MSI and MSS CRC samples. Amplification regions are shown in red; deletion regions are shown in blue; regions with no significant change are shown in green. Each column represents a single sample.

It is well known that chromosomal abnormalities differ in CRC by presence or absence of MSI. In our study, among the MSI (n = 24) and MSS (n = 62) CRC cases, there was statistical significant difference in frequency of amplification/deletion in a total of 217 genomic regions in mainly four chromosomes: chr 13, chr 17, chr 18 and chr 20 (see Table S1 for details). In MSS cases, amplification was more frequent in chr 13q and chr 20q and deletion was more frequent in chr17p and chr18 compared to the MSI cases (see Figure 12). So while looking at the association of chromosomal abnormalities with other tumor characteristics, we stratified the analyses by MSI status. We also analyzed the MSS and MSI cases combined and the results were very similar to MSS cases only (results are not shown). Detailed comparisons of all the regions with amplification and deletion in 62 MSS CRC samples with respect to different cancer and patient characteristics are presented in Table S2, S3, S4, S5, S6. A summary of these analyses is presented in the Table 3 and Table 4 for MSS and MSI cases respectively. Among the MSS cases (see Table 3), regarding the histological diagnosis, deletions at 18q12.1 and 15q15.3 were more frequently found in adenocarcinoma compared to mucinous adenocarcinoma, which supports the findings of Kazama Y et al. [27]. Regarding tumor location, some CIN were exclusive to the left sided CRC, including amplification at 20q13.2 and 13q34 regions. When tumor grading was considered, some CN changes were almost exclusive to low grade tumors, for example deletion at 18q21. Deleted18q21 region encode a total of 227 transcripts (including multiple transcripts from the same gene). Considering only genes with at least 90% overlap in the regions of aneuploidy, there were three transcripts in the deleted 18q21 region. These transcripts are from the oncogenes *RAB27B* and *CCDC68*, suggesting that deletion of this region in low-grade cases may be a protective, reactive change. Similarly, amplified region of 13q31.1 contains transcript from the gene *SPRY2*, which is associated with microtubules but may function as an antagonist of fibroblast growth factor (*FGF*) in stimulated cells. The amplification of *SPRY2* in low-grade tumors as well as lymph node negative tumors (see below) may play an important role by inhibiting the tumor growth and invasion function of *FGF*. When we looked at cytogenetic changes associated with staging, we found that amplification of 20q13 and deletion of 1p35 were more frequent in stage 1 tumors, compared to stage 2 and 3; where as amplification of 5p12 was more frequently seen in stage 2. Regarding lymph node metastasis, several amplifications (5p15.2, 5p13.1, 13q31.1 and 20q13.2) were more frequently found in lymph node negative cases compared to lymph node positive cases, possibly suggesting amplification of these regions to be protective against lymph node metastasis. We also validated the CN changes detected from this SNP array data by real-time PCR as well as by running the PCR product on Agilent BioAnalyzer 2100 using DNA 12000 chips with multiple amplicons (data not shown). We selected two genes for this validation experiment – *Cyp24A1* from 20q13.2 region, amplification of which was significantly associated with lymph node metastasis and *RAB27B* from 18q22.2 region deletion of which was significantly associated with tumor grade in combined analyses.

**Table 3:** Association of Chromosomal copy number changes with clinico-histopathological findings in MSS CRC cases (n = 62).

*Chr*	*Cytoband*	*Characteristics*	*Chromosomal Instability*			*p-value*
18	18q12.1	**Diagnosis**	Amplification	Deletion	Unchanged	0.012300
		Adenocarcinoma (n = 54)	0	13	41	
		Mucinous adenocarcinoma (n = 8)	1	0	7	
15	15q15.3	Diagnosis	Amplification	Deletion	Unchanged	0.021923
		Adenocarcinoma (n = 54)	0	6	48	
		Mucinous adenocarcinoma (n = 8)	1	0	7	
18	18q21.2	**Histological Grade**	Amplification	Deletion	Unchanged	0.029740
		Low-grade (n = 50)	0	11	39	
		High-grade (n = 12)	1	0	11	
1	1p35.3 - 1p35.2	**Tumor Stage**	Amplification	Deletion	Unchanged	0.000053
		Stage 1 (n = 11)	0	5	6	
		Stage 2 (n = 16)	0	0	16	
		Stage 3 (n = 35)	0	1	34	
5	5p12	Tumor Stage	Amplification	Deletion	Unchanged	0.000128
		Stage 1 (n = 10)*	0	0	10	
		Stage 2 (n = 16)	7	0	9	
		Stage 3 (n = 35)	1	0	34	
20	20q13.33	Tumor Stage	Amplification	Deletion	Unchanged	0.004508
		Stage 1 (n = 11)	6	1	4	
		Stage 2 (n = 16)	3	0	13	
		Stage 3 (n = 35)	4	0	31	
13	13q34	**Location of tumor**	Amplification	Deletion	Unchanged	0.020370
		Right (n = 8)	0	1	7	
		Left (n = 54)	7	0	47	
20	20q13.2	Location of tumor	Amplification	Deletion	Unchanged	0.036490
		Right (n = 8)	0	0	8	
		Left (n = 54)	20	0	34	
5	5p15.2	**Lymph node metastasis**	Amplification	Deletion	Unchanged	0.004765
		Absent (n = 27)	9	0	18	
		Present (n = 35)	2	0	33	
	5p13.1	Lymph node metastasis	Amplification	Deletion	Unchanged	0.006770
		Absent (n = 27)	7	0	19	
		Present (n = 35)	1	0	33	
20	20q13.2	Lymph node metastasis	Amplification	Deletion	Unchanged	0.014020
		Absent (n = 26)*	12	0	14	
		Present (n = 35)	6	0	29	
13	13q31.1	Lymph node metastasis	Amplification	Deletion	Unchanged	0.031390
		Absent (n = 27)	8	0	19	
		Present (n = 35)	3	0	32	

*Copy number changes for some regions could not be analyzed for one sample.

**Table 4:** Association of Chromosomal copy number changes with clinico-histopathological findings in MSI CRC cases (n = 24).

*Chr*	*Cytoband*	*Characteristics*	*Chromosomal Instability*			*p-value*
10	10p12.33	**Diagnosis**	Amplification	Deletion	Unchanged	0.025010
		Adenocarcinoma (n = 21)	1	0	20	
		Mucinous adenocarcinoma (n = 3)	0	1	2	
4	4q28.3	**Histological Grade**	Amplification	Deletion	Unchanged	0.040680
		Low-grade (n = 17)	1	0	16	
		High-grade (n = 6)*	0	2	4	

*Copy number changes for some regions could not be analyzed for one sample.

### Comparison to cytogenetic findings in other cancer

**Figure 13:**
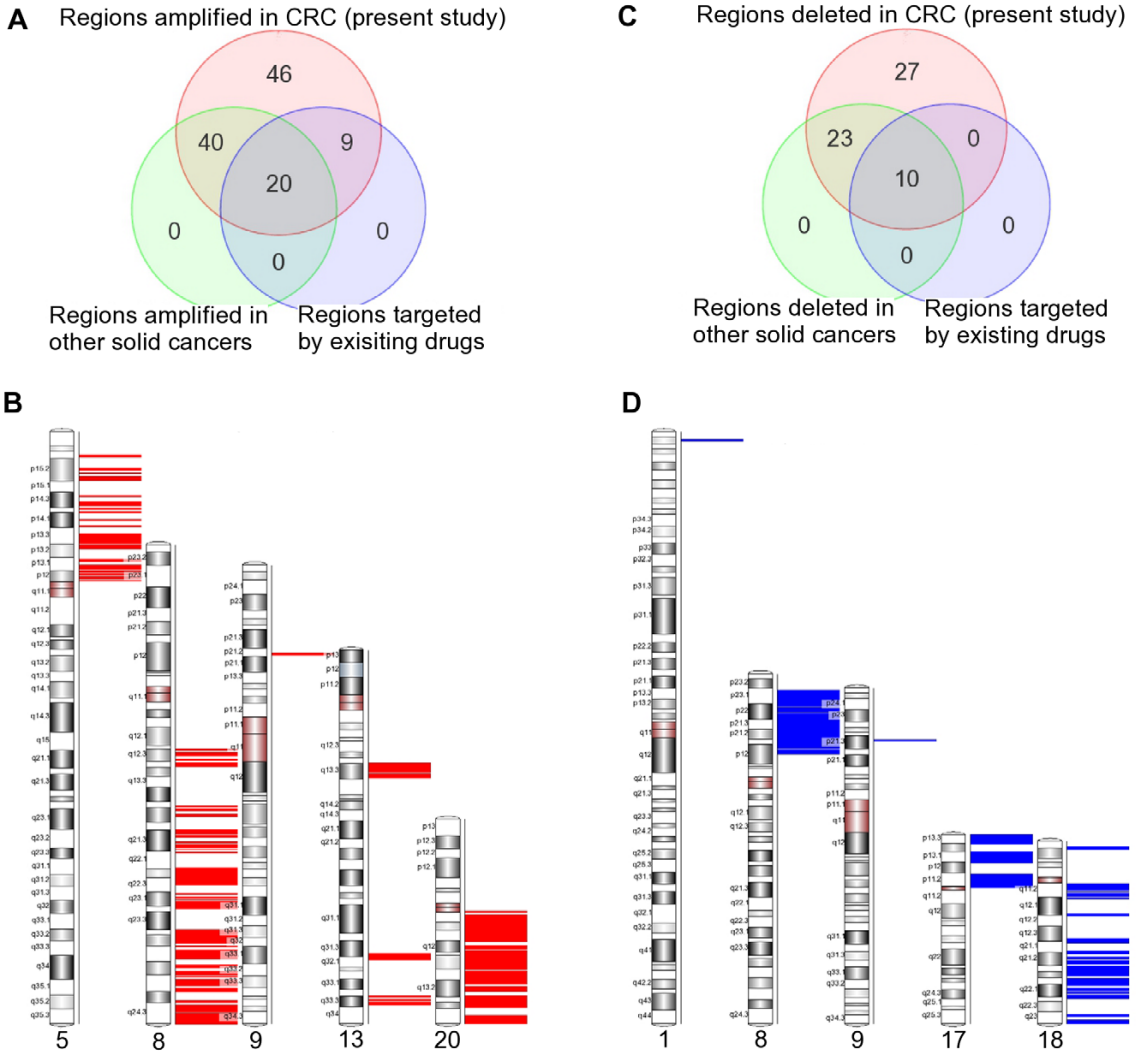
Common regions of amplification/deletion in this study and previous studies of other solid cancers. (a) Regions of amplification in our study (red circle) were also found in previous studies of other solid cancers (green circle); several are also known to be down-regulated by drugs that are approved or in development (blue circle). (b) Locations of the common regions of amplification in our study and previous studies of other solid cancers (intersection of red and green circles in Fig. 13a) are shown. (c) Regions of deletion in our study (red circle) were also found in previous studies of other solid cancers (green circle); several are also known to be up-regulated by drugs that are approved or in development (blue circle). (d) Locations of the common regions of deletion in our study and previous studies of other solid cancers (intersection of red and green circles in Fig. 13d) are shown.

We compared the genomic regions with CN change identified in our study to the genomic regions reported by other investigators to have CN change in other cancers. These studies used CGH arrays, rather than the SNP arrays used in our study. Previous studies on prostate cancer [37]–[40], lung cancer [41]–[43] and stomach cancer [44]–[46] found some common regions of amplification and deletion that we also detected in CRC in our study. The Venn diagrams in Figure 13a and 13c show chromosomal abnormalities in CRC and at least in one other cancer. There were 60 common regions of amplification and 33 common regions of deletion respectively, whose locations are shown in the ideogram of Figure 13b and 13d. It may be noted that amplifications of 5p, 8q, 20q and parts of 9p21 and 13q13 were common to multiple cancers. Similarly, deletions of 8p, 17p, a large part of chromosome 18 and portions of 1p and 9p21 were also common to multiple cancers. Therefore drugs targeted to these regions may show promising results in several different cancers.

### Therapeutic Targets Database matches

The Therapeutic Targets Database (TTD) (Version 4.3.02, July 7, 2011) [47] is a publicly available database of gene products that are known to be targeted by one or more drugs. We searched for genes that overlapped with the regions of significant amplification and deletion in our study to identify drugs that target the relevant genes. A total of 1,229 genes were identified residing in the 115 regions with amplification (100% overlapping with the amplification region in at least 10% of the CRC cases) and 920 genes were identified residing in the 60 regions with deletion (100% of the gene overlapping with the deleted regions in at least 10% of the CRC cases). The details of these regions are presented in Tables S8 (amplification) and S9 (deletion).

For genes located in amplification regions, we searched for drugs that act as antagonists, antibodies, or inhibitors. We found a total of 29 amplified regions (out of 115, see Figure 13a) harboring 39 genes that are currently being targeted by 248 different drugs of these types. It may be noted that 20 of these regions (out of 29) are also amplified in other cancers. After excluding the drugs for which approval status is not known, or discontinued in any phase of drug trial, there were a total of 69 available drugs (4 antibodies, 18 antagonists and 47 inhibitors) in different phases of trial for different purposes. The approved relevant drugs targeting these amplification regions are shown in Table 5 (for complete list, see Table S7a).

**Table 5:** Approved antibody, antagonist, inhibitor drugs targeted against genes in amplified regions in CRC.

Gene symbol	Cytoband	Drug Name	Drug Type	Drug Use
ADA	20q13.11–20q13.12	Cladribine	Inhibitor	Hairy cell leukemia
ADA	20q13.11–20q13.12	Dipyridamole	Inhibitor	Platelet inhibitor
ADA	20q13.11–20q13.12	Fludarabine	Inhibitor	Hematological malignancies
ADA	20q13.11–20q13.12	Pentostatin	Inhibitor	Hairy cell leukemia
CHRNA4	20q13.33	Pentolinium	Antagonist	Hypotension
CHRNA4	20q13.33	Trimethaphan	Antagonist	Hypertensive emergencies
CYP11B1	8q24.3	Metyrapone	Inhibitor	Cushing's syndrome (hypercortisolism)
DGAT1	8q24.3	Hesperetin	Inhibitor	High cholesterol levels
EDNRB	13q22.3–13q31.1	Bosentan	Antagonist	Pulmonary arterial hypertension
FLT1	13q12.3	Ranibizumab	Inhibitor	Age-related macular degeneration, diabetic macular edema, and retinal vein occlusion
FLT1	13q12.3	Sorafenib	Inhibitor	NSCLC; melanoma; Myelodyspalstic syndrome; AML; head and neck, breast, colon, ovarian, pancreatic, renal, hepatic cancer
FLT3	13q12.2	Sunitinib	Inhibitor	Advanced renal cell carcinoma
GHR	5p12	Pegvisomant	Antagonist	Acromegaly
HTR2A	13q14.12–13q21.1	Asenapine	Antagonist	Schizophrenia and bipolar disorder
HTR2A	13q14.12–13q21.1	Iloperidone	Antagonist	Schizophrenia
HTR2A	13q14.12–13q21.1	Sarpogrelate	Antagonist	Diabetes mellitus
SQLE	8q24.13	Butenafine	Inhibitor	Dermatologic infections
SQLE	8q24.13	Naftifine	Inhibitor	Fungal infections
SQLE	8q24.13	Terbinafine	Inhibitor	Fungal infections
SQLE	8q24.13	Tolnaftate	Inhibitor	Jock itch, athlete's foot
SRC	20q11.23	Dasatinib	Inhibitor	Chronic myelogenous leukemia, solid tumours, multiple myeloma
SRC	20q11.23	Herbimycin A	Inhibitor	Cancer
TNFSF11	13q14.11	Denosumab	Antibody	Postmenopausal osteoporosis, rheumatoid arthritis, bone metastases in prostate cancer
TOP1	20q12	Irinotecan	Inhibitor	Colorectal Cancer
TOP1	20q12	Topetecan	Inhibitor	Small cell lung cancer, second-line therapy; ovarian cancer

Likewise, for genes located in the deletion regions, we searched for drugs that act as activators, agonists, or inducers. We found a total of 10 regions of deletion (out of 60 identified in the present study, see Figure 13c) harboring 11 known genes that are currently being targeted by 29 different drugs. It may be noted that deletion in all these 10 regions are also found in other cancers. After similar filtering, as we did for regions of amplification, we found a total of 21 available drugs (1 inducer, 6 activator and 14 agonists) in different phases of trial for different purposes (for complete list, see Table S7b). Only the approved relevant drugs targeting these deletion regions are shown in Table 6.

**Table 6:** Approved agonist, activator and inducer drugs targeted against genes in deleted regions in CRC.

Gene symbol	Cytoband	Drug Name	Drug Type	Drug Use
FECH	18q21.31	Methyl aminolevulinate	Activator	Photodynamic therapy
KRT16P2	17p11.2	Griseofulvin	Inducer	Ringworm infections
LPL	8p22 - 8p21.3	Clofibrate	Activator	Dysbetalipoproteinemia
LPL	8p22 - 8p21.3	Gemfibrozil	Activator	Hyperlipidemia

Next, we identified amplified or deleted genes that are not yet targeted by any known drug in the TTD. Figure 13a shows that there are 40 more regions of amplification common to CRC and other cancers, which are not yet explored for drug development. These regions harbor a total of 207 possible gene targets (including a number of micro-RNAs and SNO) [data not shown in this paper and will be addressed in subsequent publication]. Figure 13c also shows that there are 23 more regions of deletion common to CRC and other cancers, which are not yet explored for drug development.

We also looked for agonistic drugs that target genes, amplification of which may protect against metastasis. We found a number of genes more frequently amplified in lymph node negative cases than lymph node positive cases. After searching the TTD for these genes, we found a number of agonist drugs at different stages of development against *CHRNA4, EGFL7, GRIN1, HRH3, NTSR1, PTK6* genes. As for example, Varenciline (an approved drug for smoking cessation) or AZD1446 (phase II completed for cancer and Alzheimer's disease) are agonists for *CHRNA4* (cholinergic nicotinic receptor) and can be tested whether it would be useful to prevent or delay the lymph node metastasis in CRC.

## Discussion

Our study is one of the first and largest to use such a high density SNP array on paired fresh frozen tissue (tumor and healthy) from CRC patients from a Southeast Asian population to identify CIN and correlate these abnormalities with many clinical and histopathological parameters. For the first time, we have illustrated the interpretation of CN and BAF data obtained from these SNP arrays in CRC tissue to understand the different underlying mechanisms for these CIN. However, it may be noted that SNP arrays can not detect translocations. Chromosomal abnormalities in CRC have been studied mostly by using either CGH or aCGH) [5]–[8], [10]–[12], [14]–[22]. In contrast to SNP arrays, these platforms need pure and unmixed cells for the interpretation of genomic abnormalities and do not provide BAF data. However, SNP arrays do not require pure samples, since BAF patterns clearly denote the proportion of the cell mixture (mosaicism) in the tumor itself. We have presented a few examples of this.

Using large number of paired samples, we identified a number of genomic amplifications and deletions in CRC tissue which were also reported in previous studies, with the most frequently reported gains in 20q, 13q, 7p, and 8q and losses in 17p, 18q, 8p, 4q, and 5q [5]–[8], [10]–[12], [14]–[22]. Proportion of samples showing CIN in a particular genomic region may vary from study to study depending on patient selection and the resolution of reported cytoband regions. For example, amplification in 20q13.2, in our unselected samples, was found in 24% cases, compared to 43% to 81% in other studies [5], [6], [14], [15]. It may be noted that in the series of Douglas et al. (showing gain of 20q13.3 in 81% samples) included only the CIN+ cases [6]. In our study, deletion in 18q was found in 20% cases, compared to 36% to 60% in other studies [5], [6], [14], [15]. Within our samples also, the proportions of CIN in different genomic regions were higher in MSS cases than in MSI (see Figure 12).

Our study suggests that certain chromosomal aberrations can be correlated to histopathological subtypes for CRC, cell differentiation, tumor location and lymph node metastasis, and so that this may be helpful in indicating appropriate treatments and identifying future gene targets for personalized treatments. Like a previous study [25], in left sided tumors, we also identified a deletion in 18q12.1 (23% of samples in our series). Additionally, we identified amplifications in 20q13.2 and 13q22 and deletions in 8p21.3. No significant amplifications or deletions were observed in right-sided tumors in these regions, suggesting that these changes are specific to left-sided CRC. Left- and right-sided tumors develop via different pathways: tumors with MSI are more frequently found on the right side of the colon, and tumors with CIN somewhat more frequently found on the left side [11], [28], [48]. Our study confirmed this distribution.

In our study, 87% of tumor samples were adenocarcinoma (mostly located in the left colon) and 13% were mucinous adenocarcinoma (mainly located in the right colon). We found that deletions in 15q15.3 and 15q21.1 were associated with adenocarcinoma. Kazama et al (2005) showed that mucinous adenocarcinomas are more likely to show MSI rather than CIN [27]. However, mucinous carcinomas are more likely to develop metastasis in the lymph nodes and peritoneum than adenocarcinoma [49].

As found in other studies [10], [16], [21], [26], [29], we found amplification of 20q13.2 and deletion of 17p12 to be common in early stage I CRC, suggesting that these events occur early in CRC carcinogenesis. Additionally, we found amplification of 5p15.2 and deletion of 1p36.21 to occur in 25% of stage I cases, the latter of which has been found previously to be associated with adenomas and early stage disease [29]. However, other investigators have correlated losses of 18q and 19p with stage I disease [16]. Our analysis identified several amplification events related to status of lymph node metastases in CRC. We found amplifications of 5p15.1 - 5p14.3, 13q21.2–13q21.31, 13q21.31–13q21.32, 20q13.2 and 20q13.33 to be associated with the absence of lymph node metastases. This finding suggests that amplification of these regions (and hence the over expression of the genes residing in these regions) may be a protective phenomenon against the development of lymph node metastases. Therefore, existing drugs that induce or activate these particular genes may be of clinical importance in limiting and preventing lymph node metastases. One study found decreased CN in Xp, 13q, 7q and increased CN at 11q13, 15q26, 1q23 and 8p12 to be associated with lymph node metastases. Ghadimi et al [16] found gain of 8q23–24 to be indicative of the presence of metastases in the lymph nodes which we did not find. Others have found gains in 1q23, 7p, 17q, 13 and deletions in 4p, 8p to be correlated with formation of lymph node and other metastatic lesions [24], [26], [29].

We found deletion of 18q21.1 and amplification of 13q31 in 25% and 17% of low-grade CRC cases, respectively. These changes were not found in any of the high grade cases suggesting the possibility of these abnormalities to be specific for low-grade CRC. Hermsen et al [7] found loss of 18q to be correlated with the grade of dysplasia in adenomas along with gain of 20q. Another study found gain in chromosome 1, 13, 20, 7p and 8p and loss of 4, 8p and 18q to be associated with adenoma in the progression to carcinoma [21].

### Important genes in affected cytoband regions

In TTD, we found approved antagonists and inhibitors that target particular genes found to be amplified in CRC. For example, Ranibizumab is used in age-related macular degeneration to target *FLT1* and Topetecan, a second line of therapy against small cell lung cancer is an inhibitor of *TOP1*. Denosumab is an approved drug, targeted against *TNFSF11*, and is currently being used for rheumatoid arthritis and postmenopausal osteoporosis (Table 5). The effectiveness of these drugs in CRC has not yet been investigated. Similarly, Table S6 also shows a number of inhibitors (Topotecan, Bosutinib, Sorafenib, Sunitinib, Herbimycin A etc), that are currently in trials or in use for other solid cancers, that could also be tried in CRC. Our data also suggests that there are a number of inhibitors (Cladribine, Fludarabine, etc.) currently in trials or in use for hematological oncology that may be of benefit in selective CRC patients. However, we are not in a position to comment on actual effectiveness until a clinical trial is done. It may be noted that in a previous publication, we reported some integrative analysis of CN data and gene expression data in a smaller number of CRC cases and showed that 48 up-regulated genes and 60 down regulated genes reside in genomic amplification and deletion regions respectively [50]. Our previous data also suggested that perhaps CN has a bigger influence than methylation on gene expression in CRC [50].

Our study identified amplification in 5p12 and deletion in 18p11.21 in CRC. These regions are less commonly reported in the literature. The 5p12 region harbors some important genes, for example *GHR* (growth hormone receptor), *SEPPI, PAIP1, NNT* and *FGF10* that may be related to cancer (for complete list of genes in amplification regions, see Table S8). The deleted 18p11.21 region contains important tumor inhibitory genes, for example *EPB41L3*, *L3MBTL4*, *ARHGAP28*, *PTPRM*, and *ROCK1* (for complete list of genes in deletion regions, see Table S9). Drugs that are agonists to the genes in these regions may be promising individualized treatments for patients with these specific deletions.

In contrast to the other studies, we found significant amplification of 5p15.1 - 5p14.3, 13q21.2–13q21.31, 13q21.31–13q21.32, 20q13.2 and 20q13.33 cytoband regions in the absence of lymph node metastases. At 20q13.33, there are some important genes like *DIDO1* (tumor suppressor, apoptosis inducer), *COL9A3* (major component of collagen tissue), *OGFR* (negative regulator of cell proliferation) and *ADRM1* (a plasma membrane protein which promotes cell adhesion and expression and is induced by gamma interferon). Since these particular amplifications are found in lymph node negative cases, the over expression of these gene products may in fact have a protective role against the formation of metastases and thus they may be important genes for the delay or even prevention of tumor spread. Drugs that target these genes and induce expression may help prevent tumor spread if given early enough in disease development. Another important group of genes, *CDH12* (at 5p15.1-5p14.4) and *PCDH17* (13q21.2), were found to be amplified in lymph node negative patients. *CDH12* is responsible for synthesis of cadherins, calcium dependent cell adhesion proteins, which contribute to the sorting of heterogeneous cell types. Cellular adhesion is an important function in tumor localization, or in other words may play a role in prevention of tumor spread and therefore may be another protective gene. Chalmers et al have previously suggested that cadherins are important tumor suppressor genes and are often lost in many cancers [51]. The protein encoded by *PCDH17* may play a role in the establishment and function of specific cell-cell connections in the brain, but its role in tumor tissue is not clear. However, recently Giefing et al (2011) identified *PCDH17* as a candidate tumor suppressor gene in laryngeal squamous cell carcinoma, as it was found to be significantly down-regulated in cancer cell lines [52]. Our data also supports that this gene may be a tumor suppressor and important in inhibiting tumor spread.

*AURKA* (aurora kinase A) is an important gene located at 20q13.2–20q13.33 which we found to be significantly amplified in left sided tumors compared to right sided tumors (20/67 vs. 0/19) suggesting Aurora kinase inhibitors may be effective in left sided CRC but may not be suitable for right sided CRC. From another point of view, tumors with lymph node metastasis were more often found to have amplification of the *AURKA* gene than tumors without metastasis, which suggests this gene may have a role in progression and promotion of tumor spread. In fact, this gene encodes a cell cycle-regulated kinase protein required for normal mitosis that appears to be involved in microtubule formation and stabilization at the spindle pole during chromosome segregation. Other studies also showed the similar finding [53], [54]. Aurora kinase inhibitors are an intense area of research for the development of anticancer therapies, and as such there are several drugs currently in phase I and phase II development targeting this gene. These drugs may be of future use to treat CRC patients who have this amplification to aid in the prevention of tumor progression. The *TMEM189-UBE2V1* gene (has a role in cell cycle control and cell differentiation, as well as DNA repair), *TPD52L2* gene (regulator of cell proliferation), *TP53RK* gene (stimulator of a tumor suppressor) and *PSMA7* gene (a regulator of the cell cycle) also resides in this 20q13 region. Their function indicates similar role in delaying lymph node metastasis.

Another important gene in the same region 13q31.1, *SPRY2* (protein sprouty homolog2, fibroblast growth factor antagonist), was found to be more frequently amplified in low grade (12/67 vs. 0/19), left sided (13/67 vs. 0/19) and lymph node negative tumor cases (9/38 vs. 4/48) than in high grade, right sided and lymph node positive cases. The amplification of the *SPRY2* gene in lymph node negative tumors may play an important role by inhibiting the tumor growth and invasion function of FGF and therefore could be a potential therapeutic target.

In conclusion, our study is one of the first to use a high density SNP array to analyze chromosomal abnormalities between paired tumor and healthy tissue from CRC patients and also the first to investigate this in a Southeast Asian population. We successfully identified many regions of amplification, deletion and cnLOH that are related to particular clinical and histopathological subtypes. We also identified key genes within these regions, some of which are targets for currently available drugs or drugs in the process of development suggesting that identifying chromosomal abnormalities in CRC could help in the planning of personalized treatment in future.

## Materials and Methods

The study included a total of 86 consecutive patients (M = 50, F = 36) with histologically confirmed CRC from Bangladesh meeting our study criteria. None of the patients received any radiotherapy or chemotherapy before surgery. Samples were collected from the operating room immediately after the surgical resection. For each patient, one sample was collected from the tumor mass, and another sample was taken from the resected, unaffected part of the colon about 5–10 cm away from the tumor mass. From both sites, the tissue was collected as fresh frozen, in RNAlater (RNA-stabilizing buffer, Ambion Inc.) and in Allprotect (DNA and RNA stabilizing buffer, QIAGEN) and stored at −86°C. For tumor tissue collection, the tumor specimen was sectioned and non-tumor areas were trimmed off if possible. H&E sections from the tumor samples were examined for proportion of tumor cells in each sample. Readings from at least five microscopic fields/sample examined by same histopathologist (MR) were averaged to calculate the proportion of tumor cells in a given sample which was on average 72.1% (SD 6.5%) for all 86 tumor samples with no difference between right and left sided tumors (72.1±6.31 vs. 72.1±6.67, p = 0.99). However, the proportion of tumor cells was slightly higher in MSI samples compared to MSS ones (74.6±6.24% vs. 71.2±6.48%, p = 0.029). The histopathological diagnosis was done independently by two histopathologists at Bangabandhu Sheikh Mujib Medical University (BSMMU), Dhaka, Bangladesh. For each patient we also abstracted key demographic and clinical data and tumor characteristics from hospital medical records. Written informed consent was obtained from all participants.

The research protocol was approved by the ethical review committee of BSMMU, Dhaka, Bangladesh (BSMMU/2010/10096) and by the IRB of the University of Chicago (10-264-E), IL, USA. The samples were shipped on dry ice to the molecular genomics lab at the University of Chicago for subsequent DNA extraction and high density SNP array analysis.

A total of 67 patients (M = 41, F = 34) had left sided tumors and 19 (M = 13, F = 6) had right sided tumors. The right side includes the cecum (n = 4), ascending colon (n = 7), hepatic flexure of transverse colon (n = 4) and transverse colon (n = 4) up to the splenic flexure. The left side includes the descending colon (n = 4), sigmoid colon (n = 6), rectosigmoid junction (n = 3) and the rectum (n = 54). Histopathological diagnosis showed adenocarcinoma in 75 patients and mucinous adenocarcinoma in 11 patients. Mucinous adenocarcinoma was recorded when the mucin content was more than 50% [49]. A total of 19 patients had poorly differentiated carcinoma, whereas the tumors in remaining 67 were well to moderately differentiated. TNM classification (http://www.cancerstaging.org/staging/index.html) was used for tumor staging. T1, T2, T3 and T4 represent the presence of tumor within the submucosa, with extension up to the muscular layer, involvement up to the subserous coat or extension beyond the serous coat of the colon respectively. N0 and N1 represent the tumor without or with lymph node involvement respectively. M0 represents no metastasis and M1 represents distant metastasis. Stage I, stage II and stage III are represented by T1/T2N0M0, T3/T4N0 M0 and T3/T4N1M0 respectively.

### DNA extraction

DNA was extracted from fresh frozen tissue using the Gentra Puregene kit (QIAGEN). Quality Control (QC) was performed for all samples using the Nanodrop 1000.

### Microsatellite Instability (MSI) detection

A high-resolution melting (HRM) analysis method was used for detection of two mononucleotide MSI markers – BAT25 and BAT26 [55]. A tumor was defined as MSI when it showed instability with at least one of these markers (BAT25 and BAT26), and as MSS when it showed no instability for both the markers. In this way, a total of 24 tumor samples showed MSI and all were confirmed by another relatively novel MSI marker CAT25 [56] using HRM analysis. We used published primer sequences [55], [56]. Thermocycling and melting conditions were optimized for CFX96 instrument and Bio-Rad Precision Melt Analysis software was used to identify MSI by differential melting curve characteristics. A representative example is shown in figure S1.

### Illumina SNP array

According to Illumina protocol 250 ng of tissue DNA was genotyped on either 610 Quad chips with 620,901 markers (first 24 pairs) or Cyto12 chips with 299,671 markers (remaining 62 pairs) and read on the BeadArray Reader. Each pair (normal and tumor) was placed in the same chip to avoid chip-to-chip variation. Image data was processed in BeadStudio software to generate genotype calls, B allele frequency and logR ratio. The data discussed in this paper have been deposited in NCBI's GEO and will be accessible through GEO Series accession number GSE34678.

### Statistical analysis

To compare the continuous variables (e.g. age, BMI, proportion of tumor cells, average signal intensity), we used one-way analysis of variance (ANOVA). For the categorical variables we used chi-square tests.

### Genome-wide CN analysis

BeadStudio normalized intensity values and BAF data were imported into PARTEK genomic suit [57]. Intensity data from the normal tissue was used as a reference for the corresponding CRC tissue for generating CN data for each marker (paired CN analysis). Standard Principal Component Analysis (PCA) and a sample histogram were generated as part of QC. After obtaining the paired CN data for each locus, to identify the genomic segments with amplification, normal CN or deletion, we used genomic segmentation algorithm of PARTEK. By “genomic segment” we mean a stretch of DNA segment of a particular sample showing amplification or deletion. A genomic segment with CN variation in one sample may or may not fully overlap with genomic segment in another sample. By amplification or deletion “region”, we mean the stretch of amplified or deleted genomic segment that is common to a minimum of 9 CRC samples (∼10% of the 86 samples in this paper). For the data from the 610Quad chips and Cyto12 v2.1 chips, the genomic segmentation was done with a setting of a minimum of 25 markers and 10 markers respectively, p-value threshold of 0.001 for two neighboring regions having significantly differing means. A segment was considered as amplified if the geometric mean CN was ≥2.5 and a deletion if the mean was ≤1.5. After segmentation, the data from the two types of chips were combined to get the total genomic segments from all the 86 CRC samples. Out of total 11,905 genomic segments in 86 samples we restricted the analysis for the autosomes only (10,878 segments – of which 1501 showed amplification, 1630 showed deletion and 7,747 no change in CN). The length of a genomic segment was calculated from the genomic location of the start and end SNP for that genomic segment. A total of 8,912 genomic segments (82%) were ≥500 kb size. While looking at the associations between chromosomal amplification/deletion and different CRC phenotypes findings, we restricted the analysis to these genomic segments. In this paper, we reported a genomic segment in a sample to have amplification or deletion only if it was at least 500 kb size and the geometric mean of the CN within the genomic boundary of the segment for that particular sample was ≥2.5 or ≤1.5 respectively. The phenotype of interest was tested for association with amplification/deletion status of the sample using Pearson's Chi-square test. To keep the list of associations between amplification/deletion regions and histopathological findings short, we report only the regions where the CN abnormality was detected in at least 10% of samples (Tables S8 and S9).

### Estimating mosaicism

Mosaicism between two cell populations can be estimated from SNP array data using an equation described and experimentally verified using cell lines of known aneuploidy in specially-created admixtures by Conlin et al. [58] or by Nancarrow et al [59]. In our work, we used an equation equivalent to Conlin's:
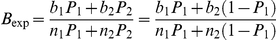
where *B*_exp_ is the expected BAF for a given SNP in admixture of two cell types; *b*_1_ and *b*_2_ are the *number* (not frequency) of B alleles for the given SNP in cell populations 1 and 2; *n*_1_ and *n*_2_ are the CN for the given loci in cell populations 1 and 2; and *P*_1_ and *P*_2_ are the proportions of cell types 1 and 2 in the admixture.

To estimate *P*_1_ of a sample, we made back-calculations of *B*_exp_ for all probable values of *b*, *n*, and *P*_1_ and reported the values for which *B*_exp_ matched the observed BAF. In practice, the observed BAF and CN limit the possible values for *b* and *n*. The macro-enabled spreadsheet used for these back-calculations is included in our supporting information.

### Therapeutic Targets Database Matching

Genes in regions of significant amplification or deletion in at least 9 samples were matched against the Therapeutic Targets Database (TTD [Version 4.3.02, July 7, 2011]) [47], [60]. The database was rebuilt using official gene symbols as the unique identifier. This was necessary for our purposes because: (a) targets in the TTD are identified by UniProt ID, a protein sequence identifier; (b) genes can be associated with multiple UniProt IDs when multiple transcripts are known. IDs were converted using the DAVID Gene ID Conversion tool [61], [62], and the database was rebuilt with our own Python scripts. When a single gene corresponded to multiple Uniprot IDs, and hence multiple TTD entries, information from those entries were combined to avoid information loss.

## Supporting Information

Figure S1 Normalized melt curves and corresponding differential curves for BAT25, BAT26 and CAT 25 amplicons. Each line represents one sample. MSS samples are shown in red. MSI samples are shown in green.
(TIF)

Table S1 Association of CN changes with MSI status.
(CSV)

Table S2 Association of CN changes with histological diagnosis.
(CSV)

Table S3 Association of CN changes with tumor location.
(CSV)

Table S4 Association of CN changes with tumor grade.
(CSV)

Table S5 Association of CN changes with tumor stage.
(CSV)

Table S6 Association of CN changes with lymph node metastasis.
(CSV)

Table S7 Therapeutic Target Database matches for: (A) antibody, antagonist, inhibitor targeting genes in the amplification regions; (B) agonist, activator and inducer targeting genes in the deletion regions.
(CSV)

Table S8 List of genes found to be significantly amplified in tumor tissue in at least 10% of samples.
(CSV)

Table S9 List of genes found to be significantly deleted in tumor tissue in at least 10% of samples.
(CSV)
