# Supplementary figures and images for: A Genome-Wide Study of Cytogenetic Changes in Colorectal Cancer Using SNP Microarrays: Opportunities for Future Personalized Treatment
Source: PLoS One. 2012 Feb 20;7(2):e31968. doi: 10.1371/journal.pone.0031968 (PMC3282791; doi:10.1371/journal.pone.0031968)

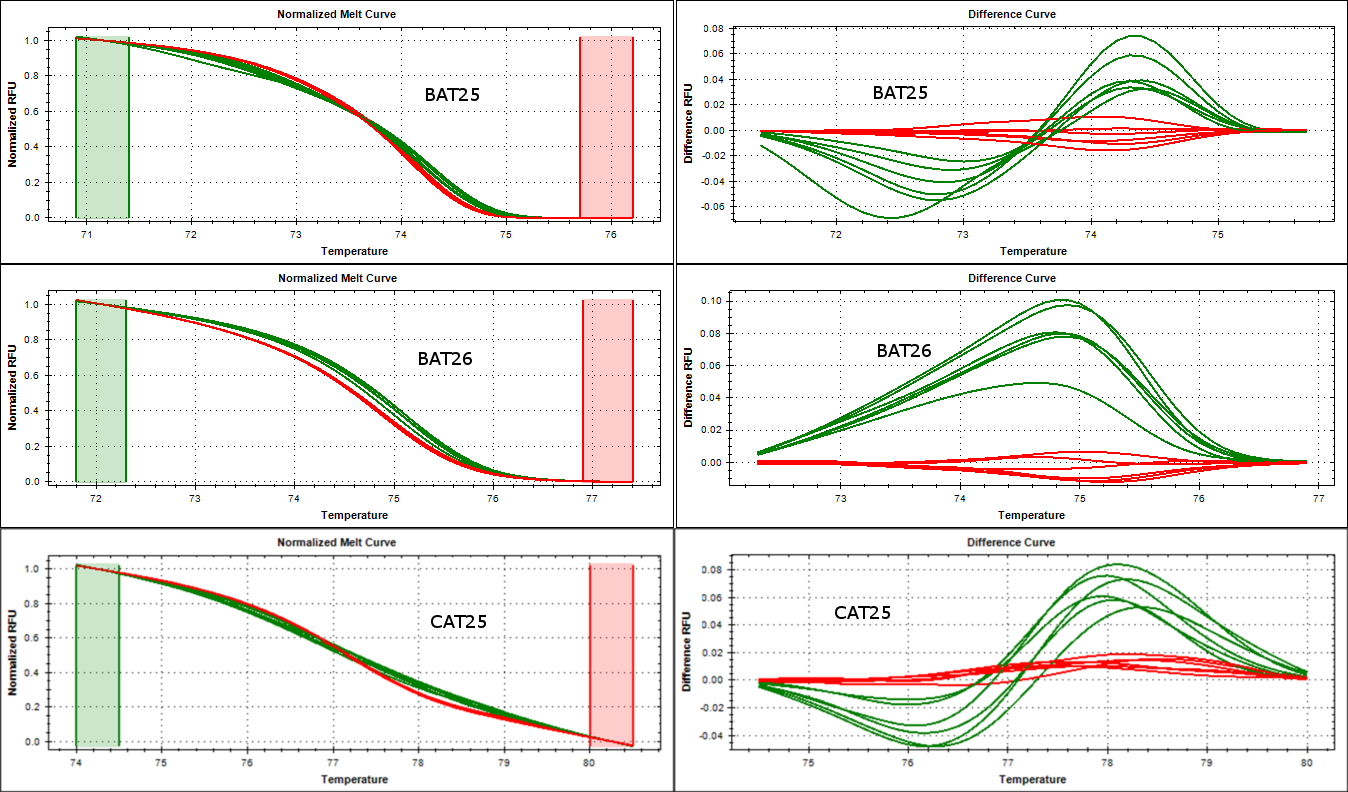

Supplement: Figure S1 — Normalized melt curves and corresponding differential curves for BAT25, BAT26 and CAT 25 amplicons. Each line represents one sample. MSS samples are shown in red. MSI samples are shown in green. (TIF) [file pone.0031968.s001.tif]
